# Integration of a task strengthening strategy for hypertension management into HIV care in Nigeria: a cluster randomized controlled trial study protocol

**DOI:** 10.1186/s13012-021-01167-3

**Published:** 2021-11-16

**Authors:** Angela A. Aifah, Oluwatosin Odubela, Ashlin Rakhra, Deborah Onakomaiya, Jiyuan Hu, Ucheoma Nwaozuru, David A. Oladele, Aina Olufemi Odusola, Ifeoma Idigbe, Adesola Z. Musa, Ayodeji Akere, Bamidele Tayo, Gbenga Ogedegbe, Juliet Iwelunmor, Oliver Ezechi

**Affiliations:** 1 NYU Grossman School of Medicine, New York City, USA; 2 Nigerian Institute of Medical Research, Lagos, Nigeria; 3 Wake Forest School of Medicine, Winston-Salem, USA; 4 Saint Louis University, St. Louis, USA; 5 Lagos State University Teaching Hospital, Lagos, Nigeria; 6 Loyola University Parkinson School of Health Sciences and Public Health, Maywood, USA; 7 Institute for Excellence in Health Equity (IEHE), NYU Langone Health, New York City, USA

**Keywords:** Practice facilitation, HIV-Hypertension integration, Implementation strategy tailoring

## Abstract

**Background:**

In regions with weak healthcare systems, critical shortages of the healthcare workforce, and increasing prevalence of dual disease burdens, there is an urgent need for the implementation of proven effective interventions and strategies to address these challenges. Our mixed-methods hybrid type II effectiveness-implementation study is designed to fill this evidence-to-practice gap. This study protocol describes a cluster randomized controlled trial which evaluates the effectiveness of an implementation strategy, practice facilitation (PF), on the integration, adoption, and sustainability of a task-strengthening strategy for hypertension control (TASSH) intervention within primary healthcare centers (PHCs) in Lagos State, Nigeria.

**Design:**

Guided by the Consolidated Framework for Implementation Research (CFIR) and the Reach Effectiveness Adoption Implementation and Maintenance (RE-AIM), this study tests the impact of a proven effective implementation strategy to integrate hypertension management into the HIV care cascade, across 30 PHCs. The study will be conducted in three phases: (1) a pre-implementation phase that will use CFIR to develop a tailored PF intervention for integrating TASSH into HIV clinics; (2) an implementation phase that will use RE-AIM to compare the clinical effectiveness of PF vs. a self-directed condition (receipt of information on TASSH without PF) on BP reduction; and (3) a post-implementation phase that will use RE-AIM to evaluate the effect of PF vs. self-directed condition on adoption and sustainability of TASSH. The PF intervention components comprise (a) an advisory board to provide leadership support for implementing TASSH in PHCs; (b) training of the HIV nurses on TASSH protocol; and (c) training of practice facilitators, who will serve as coaches, provide support, and performance feedback to the HIV nurses.

**Discussion:**

This study is one of few, if any trials, to evaluate the impact of an implementation strategy for integrating hypertension management into HIV care, on clinical and implementation outcomes. Findings from this study will advance implementation science research on the effectiveness of tailoring an implementation strategy for the integration of an evidence-based, system-level hypertension control intervention into HIV care and treatment.

**Trial registration:**

ClinicalTrials.gov (NCT04704336). Registered on 11 January 2021.

Contributions to the literature

- Few if any of the previous studies have tested practice facilitation as an implementation strategy for the integration of hypertension management into the HIV treatment in Africa.
- More importantly, reporting on the use and tailoring of implementation strategies to fit the local context has become a key priority area of research within the field of implementation science.
- Our study provides an example of how a proven effective implementation strategy, guided by two robust implementation frameworks, can be used to integrate hypertension care into the HIV treatment cascade in low-resource settings with high disease burdens.

## Background

There have been significant advances made in HIV treatment, including improved access to highly active antiretroviral treatment, which has led to increased survival among people living with HIV (PLWH) [1]. Despite these advancements, Africa accounts for 70% of the global HIV burden [2] and is overwhelmed by the increased prevalence of non-communicable diseases (NCDs) among PLWH, weak healthcare systems, and a critical shortage of the healthcare workforce [3]. While other NCDs such as cardiovascular diseases (CVD) and diabetes are common among PLWH [3, 4], hypertension remains the most prevalent and affects 14% of PLWH in Africa [5]. Hypertension among PLWH is especially concerning compared to the general population as PLWH have higher CVD mortality due to the increased burden of hypertension [6]. Given the successful rollout of HIV care programs in Africa, there is growing consensus that integration of NCD management into HIV chronic care platforms may be cost-effective for mitigating the rising burden of NCDs among PLWH [3, 7]. Additionally, a recent review noted that implementation science is critical in efforts to integrate evidence-based interventions (EBIs) into existing HIV chronic care platforms [8]. The integration of proven strategies for hypertension control, e.g., the WHO CVD Risk Package [9] into HIV chronic care platform could play a critical role in the prevention and treatment of comorbid disease burdens.

### Shifting clinical tasks for CVD treatment and management

Task shifting is the rational distribution of certain clinical services from physicians to non-physician specialists [10, 11] and has been largely established in chronic disease management including HIV [12] and CVD [11]. Shifting tasks of cardiovascular (CV) risk assessment and management of CVD risk factors like hypertension from physicians to nurses is a viable and cost-effective strategy that can be adopted for hypertension control in Africa and LMICs [11, 13, 14]. A growing consensus suggests that patients with hypertension can be treated by nurses, who provide adequate lifestyle counseling to patients [11, 13–18]. In 2008, the WHO implemented a task-shifting program for CV risk reduction and hypertension control (the WHO Package of Essential Non-Communicable Disease Intervention for Primary Care [WHO PEN]), which was designed as a cost-effective tool for systematic case management at the primary care level [9, 19]. The WHO PEN is an easy-to-follow adaptable algorithm that serves as a clinical support guideline for the assessment and management of CVD (i.e., lifestyle counseling, drug treatment, and referral protocols delivered by nurses) [19]. Its effect on blood pressure (BP) control is proven and considered a WHO Best Buy [9, 16, 20, 21]. Despite the reliability of having nurses deliver WHO PEN for CV risk assessment in primary care practices [22], its implementation within healthcare systems in Africa is non-existent.

In a cluster randomized controlled trial conducted in 32 community health centers in Ghana, our research group showed that an evidence-informed Task-Strengthening Strategy for Hypertension Control (TASSH) delivered by trained nurses, led to a 34% greater systolic blood pressure (SBP) reduction in comparison to the usual care of health insurance coverage alone [14]. Based on the WHO PEN, the TASSH intervention includes CV risk assessment, medication titration, lifestyle counseling, and patient referral [23]. Although TASSH led to significant systolic BP reduction and hypertension control, its integration into the HIV care cascade as a model for hypertension control in PLWH has yet to be evaluated. Despite the need for integrated HIV/NCD care to be recognized, evidence supporting context-specific strategies in Africa is limited [8].

### A unique opportunity for integrated care in Lagos, Nigeria

Nigeria has an established task-shifting and sharing policy, “Task-shifting and Task-sharing Policy for Essential Health Care Services in Nigeria” [10], that will make the integration of TASSH into the HIV care platform optimal and more sustainable. This policy was instituted by the Nigerian Federal Ministry of Health (FMOH) in 2014 to be implemented at national and regional levels of the healthcare system. It aims to scale up access to essential health services via efficient use of healthcare workers including nurses and community health extension workers (CHEWs). Conditions targeted include high mortality diseases like HIV, malaria, TB, maternal and infant welfare [10]. In the case of HIV care, the policy stipulates that task-shifting and sharing will avail physicians the necessary time to address more advanced clinical conditions while allowing other healthcare workers like nurses the opportunity to address more stabilized patients [10]. Provided that frontline health workers are given the necessary training and supervision; the policy promotes key tasks relating to patient adherence and medication titration to advance the treatment and care of PLWH [10]. These tasks include identifying the patient’s need for other services; making appropriate referrals; initiating treatment for acute symptoms; and conducting a routine physical examination to assess health status [10]. Although the policy has been in existence for several years, there is no evidence of its implementation as a mechanism to integrate NCD management into HIV care platforms.

### Applying a novel implementation strategy for embedding EBIs

Effective strategies for implementing evidence-based interventions (EBIs) are typically multi-level and tailored to the practice context [24–27]. In LMICs with weak healthcare systems, like Nigeria, PHCs lack the expertise needed to coordinate multilevel system changes. A practical implementation strategy to overcome this barrier is practice facilitation (PF) as it provides external expertise on practice redesign and promotes a tailored approach in implementing systems changes to improve patient outcomes [24, 28, 29]. PF involves both a *role* (practice facilitator) and a *process* for supporting primary care practices to build motivation and capacity at the systems and individual levels to improve organizational performance [30, 31]. Practice facilitators (typically nurses) are trained to work with primary care practices and help their healthcare teams develop skills they need to adopt evidence-based strategies, like task-shifting, to their practice environment [28]. A systematic review demonstrated that primary care practices with the support of a practice facilitator are about three times more likely to implement evidence-based strategies for preventive services than usual care [24]. Several studies found that the effects of PF were sustained for as long as 1 year post-intervention [32–34]. This current study is the first to integrate an evidence-based, nurse-led intervention within the HIV care cascade in primary health centers (PHCs) in Nigeria using PF as an implementation strategy.

While there is evidence of PF’s effectiveness in instituting evidence-based interventions within practice settings in high-income countries [35–38], no study has evaluated the effect of PF on the integration, adoption, and sustainability of a hypertension control intervention within PHCs in Nigeria. In this protocol, we detail our approach in using an implementation science-guided mixed-methods design to develop a context-specific PF strategy to help PHCs implement TASSH. Additionally, we document how we will evaluate the adoption and sustainability of TASSH as an integrated routine practice in PHCs within Lagos State’s primary healthcare delivery network.

## Methods

### Study design

**Fig. 1:**
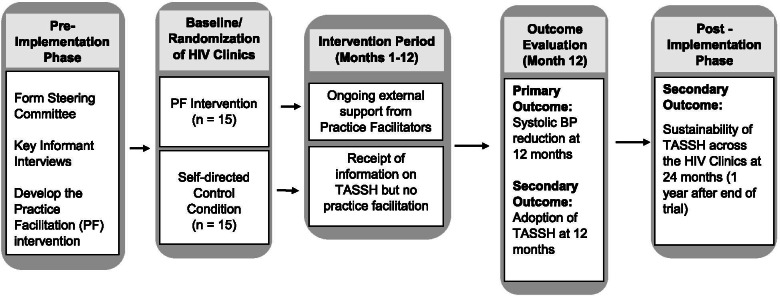
Study design

This study is being conducted in three phases (i.e., pre-implementation, implementation, and post-implementation) using a mixed-methods hybrid type II effectiveness-implementation design. Two implementation frameworks: the Consolidated Framework for Implementation Research (CFIR) and the Reach Effectiveness Adoption Implementation and Maintenance (RE-AIM) guide this study. Figure 1 provides an overview of the study design including the randomization of the 30 study sites of which 15 will be in the practice facilitation enhanced (PF + TASSH) arm and the remaining 15 will be in the self-directed control (TASSH only) arm. During the pre-implementation phase, we developed a context-specific PF strategy based on three key study-related activities: (1) data from a CFIR guided mapping exercise conducted prior to study funding; (2) discussions with Steering Committee members, and (3) rapid ethnography of the clinical workflows at the PHCs along with interviews conducted with patients and healthcare workers. The data from the CFIR mapping exercise and discussions with the Steering Committee members informed the need to conduct a rapid ethnography to better understand the clinical workflow for PLWH with hypertension as well as patients’ and healthcare providers’ perspectives on hypertension treatment within PHCs.

For the implementation phase, we will conduct a cluster randomized control trial (RCT) of 30 PHCs and 960 HIV+ patients with uncontrolled hypertension. The aim of this implementation phase is to compare the effect of PF versus a self-directed condition (i.e., receipt of the information on TASSH without PF) on systolic BP reduction at 12 months. During the post-implementation phase, the RE-AIM framework will compare the effect of PF versus self-directed condition on adoption and sustainability of TASSH at 12 and 24 months respectively. Additionally, the mediators for the adoption and sustainability of TASSH will be assessed at 12 and 24 months. This study is approved by the institutional review boards (IRB) of New York University Grossman School of Medicine and the Nigerian Medical Research Institute. The study is registered at Clincialtrials.gov (NCT04704336).

### Description of TASSH-HIV integration protocol

**Fig. 2:**
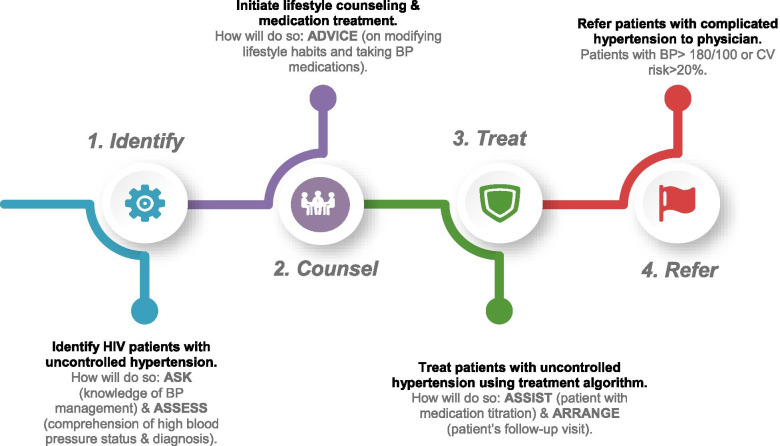
ICTR + 5A’s approach

The procedure for delivering TASSH within PHCs for PLWH is based on a four-step approach for Identifying, Counseling, Treating, and Referring (ICTR) and is defined as (i) identifying PLWH patients with uncontrolled hypertension by taking the patient’s CVD history, measuring their BP, and assessing their cardiovascular (CV) risk; (ii) initiating lifestyle counseling for PLWH with uncontrolled hypertension on adopting healthy behaviors; (iii) treating PLWH with uncontrolled hypertension by prescribing medication using Nigeria’s hypertension treatment protocol; and (iv) referring PLWH with complicated hypertension to physicians for additional care. Complicated hypertension is defined as PLWH with BP >180/110 or those with a history of stroke, diabetes, chronic kidney disease, or heart failure. Figure 2 provides an overview of the TASSH intervention and includes the protocol for the interventionists (i.e., HIV nurses and CHEWs) to deliver the intervention components using the 5A’s counseling strategy (Ask, Assess, Advise, Assist, and Arrange). The 5A’s have been used for health behavior change for patients in previous TASSH studies [14, 23] and other health outcomes such as smoking cessation [39] and weight loss [40].

### Practice facilitation (PF) implementation strategy

This study will evaluate PF’s effect on the clinical outcomes (i.e., reduction in systolic BP) for PLWH with uncontrolled hypertension as well as the adoption and sustainability of the TASSH intervention as part of Lagos State’s primary healthcare delivery network. To meet these study goals, we have developed a context-specific PF approach for integrating TASSH into HIV care at the PHCs by (a) organizing and engaging a Steering Committee consisting of key stakeholders who will provide leadership support for the adaptation and integration of TASSH into PHCs; (b) applying a train-the-trainer (TTT) model [11, 23] wherein experienced nurses from the Directorate of Nursing serve as practice outreach facilitators (POFs); and (c) training the interventionists on the TASSH protocol for BP measurement, CV risk assessment, initiation of treatment with antihypertensive medications, and referral of complicated cases.

Table 1 provides specific details of the key components of the PF strategy for the 15 study sites randomized to the PF+TASSH arm. Briefly, these PF strategy components consist of (a) POF training on using a coaching strategy developed specifically for the TASSH intervention to *Enhance, Engage, and Evaluate* [hereinafter the “3 Es”] the interventionists’ capacity to perform the TASSH duties; (b) training the interventionists on delivering the TASSH protocol i.e. ICTR + 5A’s; and (c) creating a dual community learning and engagement environment for the implementers (POFs), the interventionists, and the PLWH with uncontrolled hypertension.

**Table 1:** Overview of PF strategy components

Component	Overview
**A) Training the POFs to use the 3 E’s to support the interventionists.**	POFs will be trained to coach and support the HIV nurses in delivering TASSH as follows: • *Engage* the interventionists via monthly phone calls to address barriers in performing their TASSH duties. • *Enhance* the interventionists ability to sustain TASSH via on-site quarterly visits to observe and supervise them in their duties. • *Evaluate* the interventionist during onsite supervision and in using the learning community (WhatsApp).
**B) Training the interventionists on the TASSH protocol (ICTR + 5 A’s).**	HIV nurses will be trained to Identify, *Counsel, Treat, and Refer (ICTR)* PLWH with uncontrolled hypertension using the *5 A’s counseling strategy (Ask, Assess, Advise, Assist, and Arrange) [see Fig.**2**for more details on the ICTR+5A’s approach]*.
**C) Creating a community learning and engagement environment.**	• Developing a *community learning* environment will support learning opportunities for the POFs and the interventionists via the sharing of structured messages via WhatsApp. These messages will include content on BP measurement techniques, counseling skills, as well as the sharing of hypertension education materials. Information on tips on ICTR will also be shared. • Promoting a *community engagement* and support environment for patients by training the interventionists to identify the patient’s preferences for follow-up (i.e., frequency of reminders/phone calls) to check-up on the patient’s health and welfare.

### Conceptual frameworks

As previously noted, the study is guided by CFIR (for the pre-implementation phase) and RE-AIM (for the implementation and post-implementation phases). A robust framework for identifying and operationalizing context-relevant barriers and facilitators for intervention adaptation [41], the five domains of CFIR (i.e., intervention characteristics, outer setting, inner setting, characteristics of individuals, and the implementation process) provided pre-study data which informed the adaptation of the PF strategy for embedding the TASSH intervention within PHCs. Additional details on the use of CFIR for mapping the implementation readiness of the PHCs to integrate the TASSH intervention are published elsewhere [42].

The RE-AIM framework will inform the evaluation of the effectiveness of the TASSH intervention on BP reduction as well as the adoption and sustainability of the intervention throughout the PHCs. RE-AIM consists of five domains and has been used to measure several evidence-based interventions including diabetes self-management [43, 44], smoking cessation [44], and physical activity [43]. In this study, the five domains of the RE-AIM framework will be applied as follows: *Reach* (number of sites and people in the TASSH intervention), *Effectiveness* (impact of TASSH intervention on BP outcomes), *Adoption* (number of sites and providers using the TASSH intervention), *Implementation* (extent of intervention delivery), and *Maintenance* (sustainability of outcomes). Figure 1 illustrates the conceptual model of how this study applies CFIR and RE-AIM to integrate hypertension management into the HIV care continuum in the PHCs.

### Study setting

The study will take place in Lagos, the most populous state in Nigeria with 15 million people [45]. Implementation will occur in 30 PHCs, which are the major system for healthcare delivery including HIV treatment and care throughout Nigeria. The Nigerian Institute for Medical Research (NIMR) will select the 30 PHCs from 67 PHCs registered with the Lagos State Primary Healthcare Board with each site recruiting an average of 32 patients for a total of 960 patients for the study.

### Ethical considerations

Institutional Review Board (IRB) Approvals for this study were obtained from the NYU Grossman School of Medicine (NYUGSOM) and the Nigerian Institute of Medical Research (NIMR). The trial is also registered at ClinicalTrials.gov (NCT04704336).

### Enrollment, randomization, and allocation

Randomization will occur at the PHC level. All 30 PHCs enrolled in the study will begin the study as part of the pre-implementation phase for the first 12 months of the study, after which they will be randomly assigned to either the PF+TASSH arm (*N*=15) or the TASSH only arm (*N*=15). Once randomized, the PHCs will participate in the implementation phase (i.e., trial period) for 12 months, followed by an additional 12-month post-implementation period to assess the sustainability of the intervention. Every 6 months for 30 months, randomization will occur in 5 cohorts of 6 PHCs and will be stratified by cohort to ensure balance over time. Once recruitment is completed for each cohort, the PHCS will be randomized in a 1:1 ratio to either the PF+TASSH arm or the TASSH only arm. The sequence of randomization will be generated by a statistician and kept in a secure electronic format inaccessible to study sites, following CONSORT guidelines. Sites will be informed of their randomization group by telephone. In addition, due to the nature of the intervention, it is impossible to blind the patients, lay health advisors, and the study coordinators to the group assignment, therefore only the data analysts will remain blinded to treatment assignment until all data have been collected and the database is locked.

### Recruitment

#### Primary care practices

Primary health centers (PHCs) which are responsible for providing HIV care and treatment will be recruited and selected by the Nigerian Institute of Medical Research (NIMR), which has well-established linkages to the Lagos State AIDS Council Agency (LSACA) and the Lagos State Primary Healthcare Board with its network of 67 PHCs. Out of the 67 PHCs, we will recruit 30 PHCs. Eligibility of the 30 PHCs will include being in geographically distinct areas from one another, with an equal urban to rural mix in two arms. Once a clinic agrees to participate in the study, the clinic director will sign a memorandum of understanding (MOU) with NIMR and will be asked to identify HIV nurses to be trained.

#### Patients

We will recruit an average of 32 people living with HIV (PLWH) from each of the 30 PHCs selected from the study. Research coordinators working with the Interventionists will recruit PLWH meeting the following eligibility criteria: (a) patient receiving HIV treatment care at the selected PHC; (b) an adult aged 40 years and older; (c) have a diagnosis of hypertension with uncontrolled blood pressure, i.e., BP is 140–179/90–100 mmHg; and (d) able to provide consent. Working with the research coordinators, interventionists will identify PLWH who meet the inclusion criteria and provide a contact number to interested PLWH to follow up with the research coordinator. Research coordinators will inform interested PLWH of their 50-50 chance of being randomized to the PF+TASSH or the TASSH only arm of the study. The rationale for including only patients with uncomplicated hypertension is based on the World Health Organization’s CVD treatment guidelines [46], which mandate patients with stroke, heart failure, and kidney disease be referred to physicians for management. Patients will be excluded for the following reasons: (a) having a BP >180/110 mmHg; (b) having a history of chronic kidney disease, heart disease, diabetes or stroke, or pregnancy; (c) unable to provide informed consent; and (d) refusing to participate in the study.

#### Practice outreach facilitators

NIMR will work with the Lagos State Directorate of Nursing to recruit the practice outreach facilitators (POFs). The Directorate of Nursing is responsible for overseeing the training, planning, and evaluation of capacity-building programs for nurses in Lagos State and has a network of senior nursing officers with extensive clinical and managerial experience, including working with primary care practices as trainers. We will hire 5 senior nurses to serve as practice facilitators to the 15 HIV clinics randomized to the PF intervention group (one per 3 clinics). The decision to recruit senior nurses to train the Interventionists in implementing TASSH is a sustainable model because traditionally, in Lagos State, nursing officers are responsible for training nurses within the primary care practices. Consequently, recruiting nursing officers as POFs will be key to ensuring the adoption and sustainability of TASSH.

### Training approach

HIV nurses, CHEWs, and POFs are all trained on the TASSH protocol. POFs are further trained on coaching strategies (3 Es) to help the Interventionists implement TASSH. The duration of all training will be three full days. Following the initial training, supplemental booster training will take place every 6 months for the duration of the trial period. Table 2 provides an overview of the training approach for the POFs, HIV nurses, and CHEWs. All training sessions will be audiotaped and videotaped for future use by the trainees. All trainees will complete a pre- and post-test on all study materials such that adequate training will be based on satisfactory completion of the study materials after training. Finally, we will employ the see one, do one and teach one strategy [11, 23] to make sure that trainees acquire the necessary skills. Finally, regarding training on the referral of study participants to the PHCs, the Interventionists will be required to use electronic data capture which will be tracked for completion regularly.

**Table 2:** Training approach

*Interventionist*	*Training components*	*Training timing and frequency*
POFs (train-the-trainer model)	3E’s (to oversee intervention implementation)	Booster training sessions will occur every 6 months after the initial training date.
	TASSH Protocol (counseling using 5A’s and drug treatment)	
HIV nurses/CHEWs	TASSH Protocol (counseling using 5A’s and drug treatment)	Booster training sessions will occur every 6 months after the initial training date.
	Identify, Counsel, Treat, Refer	
	Online learning communities (using WhatsApp to increase engagement)	Ongoing

### Intervention and control conditions

For the PF+TASSH (implementation-intervention) arm, compliance with study protocol will be based on the (a) training of the Interventionists on BP measurements, counseling of eligible participants using the 5 A’s, and referral of study participants for further care based on the referral system within the PHCs; and (b) training of the POFs on using the 3 E’s to support the Interventionists. Each POF will be required to work with their assigned HIV clinics for 12 months. Over the 12-month period, each POF will conduct 13 site visits to the HIV clinics (2 in the first month, and then monthly thereafter) plus monthly peer-to-peer telephone support calls to the Interventionists.

For the TASSH only (control) arm, Interventionists will be trained on BP measurements, counseling of eligible participants using the 5 A’s, and referral of the participants for further care based on the referral system within the PHCs. The TASSH only arm will not receive practice facilitation support from the POFs. Participants attending PHC randomized to the TASSH only arm will receive standard care offered by that facility.

### Primary outcome

The primary outcome is a change in systolic BP (SBP) from baseline to 12 months of TASSH Implementation. The SBP reduction in patients will be assessed as mean change in systolic BP from baseline to 12 months. Blood pressure will be taken with a valid automated BP device as follows: at baseline, three BP readings will be taken by trained research coordinators using an automated BP monitor with the participant seated comfortably for 5 min prior to the measurements. The average of three BP readings will be used as the measure for each visit. The same procedure will be followed at the 12-month study visit. Uncontrolled BP will be defined in accordance with the Nigerian healthcare policy and WHO for CVD treatment guidelines as the average clinic systolic BP >140 mmHg or diastolic BP >90 mmHg. Adopting these guidelines currently instituted within the Nigerian healthcare system will ensure sustainability of the proposed study’s procedures.

### Secondary outcomes

There are two secondary outcomes for this study to evaluate the rate of adoption and sustainment of the TASSH intervention across the PHCs at 12 and 24 months respectively. The rate of adoption of TASSH is defined as the proportion of patients who are correctly diagnosed with hypertension, received lifestyle counseling, and antihypertensive treatment from the interventionists. For this purpose, adoption will be assessed by the following measures: (1) the number of hypertensive patients diagnosed correctly by the nurses using the WHO CVD risk assessment; (2) proportion of patients with hypertension who received lifestyle counseling from the nurses; and (3) proportion of patients for whom the interventionists initiated treatment with antihypertensive medications. In order to assess this measure, the interventionist will complete a questionnaire inquiring about the number of patients with uncontrolled hypertension who received medication treatment and lifestyle counseling. For this purpose, all interventionists will be required to keep an attendance log sheet for their patients’ visits.

Sustainability of TASSH is defined as the maintenance of TASSH uptake at the PHCs at 24 months (1 year after the end of the TASSH intervention). Sustainability will be assessed quantitatively, similar to adoption, and qualitatively, based on interviews with interventionists and clinic leadership at 24 months. Two research coordinators will conduct the interviews with two interventionists, and one key leadership personnel at each clinic. The interviews will be guided by CFIR and inquire about attitudes regarding the implementation of TASSH, barriers, facilitators, and implications for scalability. All interviews will be recorded, transcribed, and analyzed with NVIVO Version 11.

#### Mediators of TASSH

We will use several measures to assess the mediators of TASSH via self-report. The mediators are based on the constructs of the CFIR framework including inner setting characteristics of the PHCs, intervention characteristics, and implementation process measures. *Inner setting* measures include implementation climate, implementation leadership, and the organizational culture domain of the organizational social context scale. Implementation climate will be assessed with the Implementation Climate Scale that measures shared perceptions of the policies, practices, procedures, and behaviors that are expected, supported, and rewarded to facilitate the effective implementation of evidence-based practices. Implementation leadership will be assessed using the Implementation Leadership Scale (ILS)—a brief 12-item measure with four subscales: proactive Leadership, knowledgeable leadership, supportive leadership, and perseverant leadership. We will use the organizational culture domain of the Organizational Social Context Scale—a 15-item proficiency subscale—to evaluate the practice capacity proficiency level of the PHCs. Proficient organizational cultures are those characterized by shared norms and expectations that the interventionists are skilled service providers, and have current knowledge of the TASSH protocol.

*Intervention characteristics* will be measured using the evidence scale of the Organizational Readiness to Change Assessment tool, which evaluates the strength of the evidence for the proposed change or innovation [47]. For *implementation process* measures, we will use the external change agent support and the facilitation scale of the Organizational Readiness to Change Assessment (ORCA) tool [47]. The external change agent support is a 3-item tool that evaluates support provided by external facilitators, the expectations about performance and improvement, and the ways to achieve the goal of the project. The Facilitation Scale is an 8-item tool that evaluates organizational capacity to facilitate change. Table 3 provides a list of all study measures, including the data source for each and the timing of administration.

**Table 3:** Study measures

Construct	Measures	Data source	Timing of administration
**Pre-implementation phase**			
Intervention characteristics	• Organizational readiness to change (12-item Evidence Scale)	• Steering committee and staff surveys	Baseline
Inner setting	• Implementation Climate Scale • Implementation Leadership Scale • Organizational Culture domain of the Organizational Social Context Scale	• Semi-structured interviews with Key Stakeholders • Steering committee and staff surveys	Baseline
Implementation process	• External Change Agent Support tool (3-item tool) • Organizational Readiness to Change Assessment (Facilitation Scale-8 item)	• Steering committee and staff surveys	Baseline
**Implementation phase**			
Systolic BP reduction (primary outcome)	• Automated BP Monitor (according to WHO guidelines) • Participant characteristics	• Clinic patient medical records	Baseline and 12 months
Adoption of TASSH (secondary outcome)	• CVD Risk Assessment • Lifestyle Counseling • Medication Titration	• Nurse interviews and questionnaires • Attendance log sheet patient visits	12 months
**Post-implementation phase**			
Sustainability of TASSH (secondary outcome)	• CVD Risk Assessment • Lifestyle Counseling • Medication Titration	• Nurse interviews and questionnaires • Attendance log sheet patient visits	24 months

### Statistical approach

#### Power considerations and sample size

The TASSH intervention will be delivered through group sessions at clinics and thus the appropriate design is a cluster-randomized trial where the unit of randomization is the clinic. There are two important study design features: the number of PHCs, and the number of participants per PHCs. Specifically, the participants will be nested (grouped) within the PHCs. From our previous work implementing TASSH, we can expect an effect size of approximately 3.5 mmHg, with a standard deviation of approximately 12 mmHg; this leads to a standardized effect size of approximately 0.3. Thus, we estimate sample sizes for a range of small to moderate effect sizes (*d* = .30 to .50) for this intervention, assuming an average cluster size of about 30 participants per PHC, and an ICC of between .02 and .05 at the significance level of 0.05 (two-sided test). Based on these assumptions and calculations we have established recruitment goals of 30 clinics (15 in each arm) with 32 participants per PHC. This yields a total sample size of 30 × 32 = 960 participants, providing more than 90% power to detect a small effect size of 0.3 with a conservative assumption of ICC = 0.04. This sample size calculation has included adjustment for up to 20% attrition of the study sample.

#### General analytical approach

All data will be summarized and presented in tabular and graphical format, using means, standard deviations, medians, and ranges for continuous variables and proportions for categorical variables. We will assess balance for baseline characteristics according to randomized treatment assignment. Analyses will be conducted according to the principle of intention-to-treat, in which every PHC is analyzed according to the assigned intervention, regardless of the condition received.

#### Primary outcome analysis

The analysis will consist of a repeated-measures mixed-effects model for systolic BP (SBP), with fixed effects for time and intervention arm, and random effects for PHC. We will include an interaction term for the intervention arm and time; this, if non-zero, will indicate that the degree of change of SBP over time differs for those in the practice facilitation intervention compared to those in the self-directed condition. We will also assess whether adjustment for any baseline characteristics is necessary, including such adjustments based on the change-in-estimate criteria.

#### Secondary outcomes analysis

We will evaluate the difference between the intervention arms in the adoption and sustainability of the TASSH program at 12 and 24 months. We will use a multi-level mixed model using an unstructured covariance matrix, with the outcomes defined as the proportion of participants within a PHC who were diagnosed with hypertension, received lifestyle counseling and treatment for hypertension at 12 months (adoption), and the proportion of participants within a PHC who have received screening, counseling and hypertension treatment at 24 months (sustainability). For adoption, the analysis will have one within-person factor: time (baseline and 12-month coded naturally as months [0 and 12]) and one primary between-patient factor (randomization group dummy coded as 0 = Self-directed Condition and 1 = Practice Facilitation). Fixed effects will be specified for time, randomization group, and their interaction effect (group by time). The outcome measure will be a composite index for the adoption of TASSH (defined as patients diagnosed with hypertension, who received lifestyle counseling and treatment for hypertension by the interventionists).

Additionally, the interventionists will be nested within PHCs creating a 3-level analytic model (observations nested within interventionists nested within PHCs). Random effects will be specified for PHCs and interventionists, adjusting for the clustering of measures within interventionists and interventionists within a PHC. Multi-level modeling software (SAS, Version 9, PROC MIXED) will be used to compute full information maximum likelihood (FIML) estimates of the model parameters. The PROC MIXED procedure will use an error structure that allows for the possibility of group differences in the error variances at 12 months and the serial correlations of the baseline with the 12-month outcomes. For sustainability, the analysis will be repeated as described above for adoption but will be evaluated at 24 months instead of 12 months. Adoption at 12 months will be assessed using questionnaires completed by the interventionists and sustainability at 24 months will be assessed with site interviews and visits. Levels of adoption and sustainability will be compared between the group that randomized to the PF intervention and the group randomized to the self-directed condition. The qualitative components of sustainability at 24 months will be assessed using interviews with the interventionists and clinic leadership. These interviews will be recorded, transcribed, and entered into NVIVO Version 11 for analysis.

#### Mediators’ outcome analysis

We will evaluate the mediators of adoption and sustainability of the PF intervention at 12 and 24 months. In particular, we will assess the extent to which inner setting variables (e.g., implementation leadership, implementation climate, and organizational culture) affect the degree of adoption of TASSH and its sustainability at 24 months. We will pay particular attention to the pathways via which this occurs. We will estimate a just-identified path model using the robust weighted least squares estimator to investigate relationships among the theoretical mediators of implementation climate, implementation leadership, organizational culture, organizational readiness to change, and external change agent support. Based on our conceptual model, we will test the direct effects from the theoretical constructs to the adoption components (individually). In addition to the direct effects, the indirect effects from each variable to adoption via inner setting variables will be estimated as the product of component direct effects and tested using bootstrapped 95% confidence intervals. Finally, we will estimate the direct effects of the predicted model of adoption on SBP reduction. Predicted probabilities of the adoption and sustainability outcomes and SBP will be calculated from path model coefficients to elucidate the magnitudes of direct and indirect effects.

#### Handling missing data

Although we will attempt to retain as high a fraction of participants as possible, we acknowledge that some attrition is likely, leading to missing outcome values. In the instance of missing data, the generalized linear mixed models proposed for the primary and secondary analyses incorporate an assumption of data that are missing at random (MAR) i.e., the likelihood of a value being missing depends on observable characteristics (e.g., sex or age). In sensitivity analyses, we will assess the impact of different assumptions about the missing data mechanism and will determine the robustness of trial results to these different assumptions. We will consider the use of multiple imputations of missing data as an alternative sensitivity analysis [48].

#### Qualitative analysis

For qualitative data analysis, semi-structured and user-testing interviews will be transcribed and entered into the qualitative software, NVivo, version 11, for organization and management of the qualitative data obtained. We will use the framework approach to qualitative data analysis, a 5- step process: (a) familiarization, (b) developing a theoretical framework, (c) indexing, (d) summarizing data in an analytical framework (in this case using CFIR constructs), and (e) data synthesis and interpretation. Following this framework, data will be independently coded by 2-3 experienced research staff to reduce the potential for bias. Inter-rater reliability will be determined and discrepancies in coded data will be resolved by consensus.

All transcripts will be coded into concepts reflecting the aims of the pre-implementation phase. For example, responses will be coded according to intervention characteristics (e.g., core elements of TASSH) likely to influence its adoption in PHCs. The identified concepts will be grouped into categories and themes uniting the categories determined. A detailed analysis of the interviews should generate a conceptual model of the barriers and facilitators of TASSH uptake and domains of a PF strategy tailored to the Nigerian healthcare system.

## Discussion

Access to highly active antiretroviral treatment led to increased survival of PLWH in Africa [1] and has now placed PLWH at an increased risk for CVD. To prevent a reversal of the gains made in HIV treatment and access, strategies to control hypertension in PLWH are crucial. However, the acute shortage of healthcare workforce limits the effective reduction of hypertension-related morbidity among PLWH. Task shifting of duties from physicians to nurses may mitigate this systems-level barrier to hypertension control. For countries like Nigeria—which faces acute healthcare workforce shortage and is now experiencing an increase in hypertension prevalence among PLWH [49]—strategic, evidence-informed implementation strategies for embedding effective healthcare workforce strengthening interventions will be critical to addressing this dual disease burden.

This study is one of few studies in Nigeria, let alone Africa, to address the burden of comorbid hypertension-HIV and the acute shortage of healthcare workers by applying an implementation science-supported strategy (PF) for integrating hypertension-focused intervention for PLWH. Consequently, there are several novel aspects of this study in terms of its likelihood to exert a sustained influence on the research field, particularly implementation science. First, this study combines well-established implementation science frameworks (CFIR and RE-AIM) into an intervention to assess the influence of inner setting and provider characteristics on the adoption of task-shifting strategies for hypertension control in PLWH. While extant literature in LMICs shows that the uptake of evidence-based interventions is a desired outcome of implementation research, there is currently limited evidence of such interventions in Africa [50, 51], especially those targeted at hypertension control in PLWH.

Second, considering the growing interest in utilizing robust research methods to identify barriers and facilitators of translating evidence-based interventions into practice, this study will advance the field by using a robust implementation strategy (PF) to provide contextual support for integrating hypertension control into HIV care in PLWH. Finally, although the effectiveness of task-shifting strategies for HIV care in Africa is well-established [14], integration of hypertension management into HIV care platforms using this strategy has not been evaluated. This is the only study we are aware of that will evaluate the integration of a task-shifting strategy for hypertension control into PHCs in Lagos. The innovation of the proposed study is unlike a previous study in Ghana [52] as it will evaluate the effect of a systems-level practice facilitation strategy on hypertension control in PLWH. If successful, its findings will provide evidence for policymakers to adopt TASSH as routine practice for HIV clinics in Nigeria and similar LMICs. While our previous study demonstrated the effectiveness of TASSH, the current study will evaluate its implementation by indigenous staff and integration to PHCs in Lagos for the treatment and care of hypertension among PLWH.

## Limitations

There are several methodological challenges that this study may encounter over the course of its implementation. First, while medication titration is included in this study as part of the task-shifting duties for the interventionists, we do not provide medication for patients nor do we offer access to hypertension medications. Secondly, the sample size calculation is based on an average cluster size (number of participants enrolled in each PHC) without considering potential discrepancies in cluster sizes. In practice, the size of the PHCs quantified as the number of PLWH seen at each clinic has high variability. In order to balance the enrollment burden across different PHCs, we employ the probability population to size method to determine the number of participants to be enrolled per PHC. This would potentially lead to a reduction of efficiency [53].

## Conclusion

The current study will have a high impact on the field of implementation science in Africa for two reasons. First, to ensure sustainability, this study will integrate an evidence-based intervention for hypertension control into the vast HIV chronic care platform based on Nigeria’s task-shifting and task-sharing policy for essential medical services. Second and more importantly, we chose a practical implementation strategy that is based on well-established implementation frameworks, CFIR and RE-AIM, that have not been tested in PLWH, thus assuring its influence on the field of implementation science in Africa.

If successful, findings from this study will demonstrate the effectiveness of strengthening the capabilities of PHCs as a platform for integrated HIV/NCD care in Nigeria, where the HIV burden remains high. Additionally, task shifting of primary care duties from physicians to nurses using a PF strategy will ease the acute shortage of healthcare workers on the already weak health systems. Finally, findings will provide the evidence base for the adoption of similar strategies to reduce the burden of other CVD risk factors like diabetes, and other NCDs in PLWH.

## Data Availability

Not applicable.

## Abbreviations

CFIR: Consolidated Framework for Implementation Research
CHEWs: Community health extension workers
CV: Cardiovascular
CVD: Cardiovascular disease
EBIs: Evidence-based interventions
FIML: Full information maximum likelihood
FMOH: Federal Ministry of Health
ICTR: Identifying, Counseling, Treating, and Referring
IRB: Institutional review boards
LSACA: Lagos State AIDS Council Agency
MAR: Missing at random
MOU: Memorandum of understanding
NCDs: Non-communicable diseases
NIMR: Nigerian Institute of Medical Research
NYUGSOM: NYU Grossman School of Medicine
ORCA: Organizational Readiness to Change Assessment
PF: Practice facilitation
PLWH: People living with HIV (PLWH)
PHCs: Primary healthcare centers
POFs: Practice outreach facilitators
RE-AIM: Reach Effectiveness Adoption Implementation and Maintenance
SBP: Systolic blood pressure
TASSH: Task-strengthening strategy for hypertension control
WHO: World Health Organization
